# Integrative Approach to Analyze Biodiversity and Anti-Inflammatory Bioactivity of *Wedelia* Medicinal Plants

**DOI:** 10.1371/journal.pone.0129067

**Published:** 2015-06-04

**Authors:** Wen-Ching Lin, Chih-Chun Wen, Yung-Hsiang Chen, Pei-Wen Hsiao, Jiunn-Wang Liao, Ching-I Peng, Ning-Sun Yang

**Affiliations:** 1 Agricultural Biotechnology Research Center, Academia Sinica, Taipei, Taiwan; 2 Institute of Plant Biology, National Taiwan University, Taipei, Taiwan; 3 Graduate Institute of Veterinary Pathology, National Chung Hsing University, Taichung, Taiwan; 4 Biodiversity Research Center, Academia Sinica, Taipei, Taiwan; University of Naples Federico II, ITALY

## Abstract

For the development of “medical foods” and/or botanical drugs as defined USA FDA, clear and systemic characterizations of the taxonomy, index phytochemical components, and the functional or medicinal bioactivities of the reputed or candidate medicinal plant are needed. In this study, we used an integrative approach, including macroscopic and microscopic examination, marker gene analysis, and chemical fingerprinting, to authenticate and validate various species/varieties of *Wedelia*, a reputed medicinal plant that grows naturally and commonly used in Asian countries. The anti-inflammatory bioactivities of *Wedelia* extracts were then evaluated in a DSS-induced murine colitis model. Different species/varieties of *Wedelia* exhibited distinguishable morphology and histological structures. Analysis of the ribosomal DNA internal transcribed spacer (ITS) region revealed significant differences among these plants. Chemical profiling of test *Wedelia* species demonstrated candidate index compounds and distinguishable secondary metabolites, such as caffeic acid derivatives, which may serve as phytochemical markers or index for quality control and identification of specific *Wedelia* species. In assessing their effect on treating DSS induced-murine colitis, we observed that only the phytoextract from *W*. *chinensis* species exhibited significant anti-inflammatory bioactivity on DSS-induced murine colitis among the various *Wedelia* species commonly found in Taiwan. Our results provide a translational research approach that may serve as a useful reference platform for biotechnological applications of traditional phytomedicines. Our findings indicate that specific *Wedelia* species warrant further investigation for potential treatment of human inflammatory bowel disease.

## Introduction

As complementary and alternative medicine (CAM) is increasingly recognized for its potential in evidence-based public healthcare applications, research into the safety and efficacy of medicinal plants, has become vital for public health. A number of reputed medicinal herbs that are likely to be clinically useful have been proven to be highly toxic if used incorrectly, and medication errors, misuse of plant species with similar appearances, and adulterants or consumption of unknown and complex herbs in various clinical situations may also hinder toxicology and effectiveness analyses [1]. The key to herbal medicine safety is positive verification and guaranteed purity of medicinal plants and the derived extracts. To date, various macroscopic and microscopic techniques, molecular marker authentication, metabolite profiling and metabolomics analyses have been employed, often individually or non-systematically, for verification and validation of putative medicinal plants in an effort to authenticate medicinal herbs [2–4]. Chemical chromatographic fingerprinting by high performance liquid chromatography (HPLC) [5] and ultra performance liquid chromatography coupled with electrospray ionization and quadrupole time of flight mass spectrometry (UPLC-ESI-Q-TOF-MS) [6,7], in combination with principal component analysis (PCA) [8] have been proven to be a powerful platform for metabolomics studies, as they not only are useful for comparing the chemical profiles of herbal medicines, but also for identifying chemical markers or indices. In this study, we intentionally combined all of these various technology systems mentioned above for and integrated investigation for phytomedicine use of a specific medicinal herb, *Wedelia chinensis*, that we recently demonstrate to confer strong anti-colitis activity in experimental mouse system [9].

Inflammatory bowel disease (IBD), with a high incidence worldwide, is a result of chronically relapsing idiopathic inflammation of the gastrointestinal tract [10,11]. Patients with IBD typically also run a high risk of the disease developing into colorectal cancer due to chronic damage and inflammation in the colon and tissues of the rectum [12]. The DSS-induced mouse colitis model is well accepted for studying IBD due to its resemblance to human ulcerative colitis [13,14]. Drugs for treating IBD including 5-aminosalicyclic acid, sulfasalazine, and others [15,16]; however, IBD patients usually need long-term and repeated treatment, involving potentially toxic drug doses [16,17]. Because of such side-effects or/and lack of effectiveness of standard western medicine therapies, many IBD patients turn to complementary and alternative medicine (CAM). Herbal therapy is the most common type of CAM used for gastrointestinal disorders [18–21], but so far there have been limited reports of efficacious CAM therapeutic strategies or safe phytomedicines to remedy IBD.

*Wedelia chinensis* belonging to family Asteraceae (sunflower family) is commonly consumed orally. It is often infused with hot water to make an herbal tea. Such herbal tea drinks, as cold beverages, are readily available commercially in Taiwan, China Mainland and other Asian countries. We revealed in our previous study [9] that the crude plant extract of *W*. *chinensis* can confer a specific anti-inflammatory effect on dextran sulphate sodium (DSS)-induced murine colitis. *In vivo* treatment with the hot water extract of *Wedelia chinensis* can effectively suppress the DSS-induced increase in expression of macrophage-derived/Th1 (TNF-α, IFN-γ) and Th17 (IL-17) cytokines, but has no effect on the expression of Th2 (IL-4) cytokines. According to the *Flora of Taiwan* (2nd edition), four *Wedelia* species and two additional varieties grow in Taiwan [22]. Since taxonomists can make use of different parameters and resolutions for the definition of a species or in defining speciation, we were in fact able to find a range of different classifications of these six *Wedelia* species in the literature. For example, a number of different reports reduced *W*. *trilobata* to the genus *Sphagneticola*, and *W*. *biflora* to the *Wollastonia* species [23–25]. According to the current taxonomical classification, *Sphagneticola trilobata* and *Wollastonia biflora* are the academically accepted taxonomic names, while *Wedelia biflora* and *Wedelia trilobata* are synonyms. *W*. *biflora*, *W*. *prostrata* and their varieties were also reduced to the genus *Malanthera* [26]. These six *Wedelia* species have similar appearances, particularly *W*. *chinensis* and *W*. *trilobata*. These plants often bewilder the general public and herbal medicine users because of the lack of distinction in their common trivial names and their similar morphology. Different species are sold in herbal markets in Asia, not only as an ingredient in herbal tea, but also as an anti-inflammatory tonic or medicinal food. The Zhongyao Da Cidian (Encyclopedia of Chinese Materia Medica), the most comprehensive work on Chinese Materia Medica since the Bencao Gangmu (Compendium of Materia Medica), records that *Wedelia* plants confer benefit against dysentery, an inflammation of the intestines especially the colon [27]. There is, however, little or no information about which species or cultivar strains of *Wedelia* have the potency or specificity to serve as desirable medicinal food, especially for control of various inflammatory activities related to human health. Therefore we used a DSS-induced mouse colitis model, which is well-established in our laboratory and has many similarities to human ulcerative colitis, as a biotechnological platform for systematic analysis of the anti-inflammatory activities of the six different *Wedelia* species/varieties found in Taiwan.

In this study, our primary goal was to establish an effective biotechnological approach and platform through which morphological, histological and phytochemical profiles and biodiversities are comparatively studied among different *Wedelia* species. Effects were also made to evaluate the safety and potential medicinal efficacy of these traditional medicinal herbs. To this end, we integrated several authentication methodologies, including macroscopic and microscopic examination, molecular identification, and metabolomics analysis to identify and characterize the six medicinal plants from the *Wedelia* genus commonly found in Taiwan. To evaluate the anti-inflammatory bioactivity and therapeutic efficacy of these *Wedelia* species, we used a DSS-induced acute murine colitis system as an animal model. We then explored the possible correlation between our metabolite fingerprinting and phytochemical analyses data (obtained with principle component analysis [PCA]), and the bioactivity results obtained from *in vivo*, anti-colitis animal experiments. Our findings provide useful baseline and regional speciality information on the biodiversity of the *Wedelia* genus plants. Specific plant secondary metabolites that may confer potent bioactivities in both mammalian and plant systems are discussed. The possible application of our findings to systematic exploration of plant secondary metabolites as phytomedicines present in reputed, traditional medicinal plants is contemplated.

## Methods and Materials

### Materials

Aerial plant parts of four different species and two additional varieties of *Wedelia* were collected from the northern counties of Taiwan. Some *Wedelia* plant samples were kindly provided by the Miaoli District Agricultural Research and Extension Station (MDARES), National Museum of Natural Science (NMNS) and I-Shun herbal shop in Taiwan (see details described in Table 1). Each of these species was initially authenticated by taxonomy expert Professor Ching-I Peng (Research Center for Biodiversity, Academia Sinica). Voucher specimens were deposited in the HAST Herbarium, Biodiversity Research Museum, Academia Sinica, Taiwan.

**Table 1:** Taiwan local collection sites of *Wedelia* species.

Species	Collection localities		Date
WC	National Taiwan University	Da’an Dist., Taipei City	05.07.2010
			07.09.2010
	Lailai	Gongliao Dist., New Taipei City	03.23.2010
			04.28.2010
			07.12.2010
	Bitou	Ruifang Dist., New Taipei City	07.10.2010
	Nanya	Ruifang Dist., New Taipei City	07.10.2010
	I-Shun herbal shop	Banqiao Dist., New Taipei City	10.15.2010
			12.31.2010
			07.04.2011
			08.09.2011
	MDARES*^1^	Gongguan Township, Miaoli County	08.03.2011
			09.05.2011
WT	Bat Park	Ruifang Dist., New Taipei City	03.01.2010
	Academica Sinica	Nangang Dist., Taipei City	07.07.2010
			08.23.2011
	Bitou	Ruifang Dist., New Taipei City	07.10.2010
	Nanya	Ruifang Dist., New Taipei City	08.25.2011
	NMNS*^2^	North Dist., Taichung City	08.26.2011
WB	Bat Park	Ruifang Dist., New Taipei City	03.23.2010
	Nanya	Ruifang Dist., New Taipei City	07.18.2010
			08.25.2011
Species	Collection localities		Date
	Jinshawan	Gongliao Dist., New Taipei City	07.18.2010
			09.07.2011
	Fulong	Gongliao Dist., New Taipei City	04.28.2010
			07.10.2010
	MDARES*^1^	Gongguan Township, Miaoli County	08.03.2011
			09.05.2011
WP	Fulong	Gongliao Dist., New Taipei City	03.23.2010
			04.28.2010
			07.10.2010
			08.25.2011
			09.07.2011
	MDARES*^1^	Gongguan Township, Miaoli County	08.03.2011
			09.05.2011
WBR	Jinshawan	Gongliao Dist., New Taipei City	07.18.2010
WPR	Fulong	Gongliao Dist., New Taipei City	03.23.2010
			04.28.2010
			07.10.2010
			08.25.2011
			09.07.2011
			09.19.2011

*^1^ MDARES: Miaoli District Agricultural Research and Extension Station.

*^2^ NMNS: National Museum of Natural Science.

### Extraction

Fresh aerial plant parts, leaves and stems, of *Wedelia* plant were combined with sterilized Milli-Q water at a ratio of 1:10, and then boiled to one-third of the original volume. Extracts were filtered using a glass funnel (Buchner GF16-25G3, Iwaki TE-32 Pyrex) and filter paper No. 2, 90 mm (Toyo Roshi Kaisha). Finally, the extracted samples were concentrated using a rotary evaporator (Büchi, Rotavapor R-200), and subsequently lyophilized using a freeze-drying system (Freezone 4.5, Labconco).

### Tissue sectioning and histological analysis

Transverse sections of the plant stem and murine colon tissues were prepared using a Leica RM2125RT microtome (Leica Instruments, Nussloch, Germany). Photomicrographs were taken using an AxioCam HRc videocamera (Zeiss) connected to an optical microscope (Zeiss Imager Z1) using AxioVision Rel. 4.8 software. A stereomicroscope (SteREO Lumar V12, Zeiss) equipped with AxioCam MRc and AxioVision Rel. 4.8 software was also used to photographically record test specimens.

### Safranin-fast green staining of plant tissue sections

The fresh stems of six species/variants of *Wedelia* were cut into appropriate segment sizes and fixed in FAA (formalin, acetic acid, and 70% ethanol mixed at a ratio of 1:1:18, respectively). Tissue samples were processed through a gradual ethanol series (from 30% to 100%) and dimethylbenzene for specimen dehydration [28], then buried in paraffin blocks according to the technique described by Ruzin (1999). Subsequently, tissues were sectioned with a microtome to a suitable thickness (10 μM) and stained with safranin-fast green (1% Safranin O in methylcellosolve, 95% ethanol and water at a ratio of 2:1:1, respectively), then in 1% NaOAc and 2% formalin solution, followed by 0.5% Fast Green FCF in methyl cellosolve, 100% ethanol and clove oil at a ratio of 1:1:1, respectively. Finally, samples were mounted in Histokitt mounting medium (Assistant) for observation.

### DNA extraction

The CTAB method [29], with some modification, was employed for DNA extraction. Small quantities (50–100 mg) of fresh leaves were frozen in liquid nitrogen and ground to fine powders. The powder derived from 50–100 mg fresh leaves was added to 0.7 mL of extraction buffer [2% (w/v) cetyltrimethylammonium bromide (CTAB), 0.1M tris-HCl (pH 8.0), 20 mM EDTA (pH 8.0), 0.2% (w/v) PVP-40, 1.42 M NaCl, 5 mM ascorbic acid, 0.02% 2-mercaptoethanol]. Subsequently, 1 μL RNase A (10 mg/mL) was added to the test sample and incubated at 65°C for 30 min. The tissue homogenate was then centrifuged at 13,000 rpm for 10 min. DNA was extracted using 0.8 volume of phenol/chloroform/isoamyl alcohol (IAA) at a ratio of 25:24:1, respectively, and 0.8 volume chloroform/IAA (24:1). After centrifugation at 13,000 rpm, the supernatant was purified using a DNeasy Plant Mini Kit (Qiagen), according to the manufacturer’s instructions.

### Amplification and analysis of ITS sequences

Plant genomic DNA of six *Wedelia* species were used as templates to amplify the ITS sequences, by employing 20 pmol each of the primers TCM-1 (5′-CACAAAGCCCGTCGCTCCTACCGA-3′) and TCM-2 (5′-ACTCGCCGTTACTAGGGGAA-3′) [4] in a 20 μL solution containing 100 ng of genomic DNA, 1× reaction buffer, 250 μM dNTP, and 1 unit Phusion Hot Start high-fidelity DNA polymerase (Fermentas). PCR assays were conducted using a Biometra T3000 Thermocycler: 98°C for 1 min, 30 cycles of 98°C for 30 sec, 60°C for 1 min and 72°C for 1 min, and 72°C for 10 min. The products were cloned into pJET1.2/blunt cloning vector (Fermentas). Rapid Plasmid Miniprep System (Geneaid) was then used for plasmid purification. Purified DNA plasmids were sequenced using an ABI Prism 3730 DNA Analyzer. Sample sizes: (1) *Wedelia chinensis*: n = 39; (2) *Wedelia trilobata*: n = 18; (3) *Wedelia biflora*: n = 27; (4) *Wedelia prostrate*: n = 21; (5) *Wedelia prostrata var*. *robusta*: n = 3; (6) *Wedelia prostrata var*. *robusta*: n = 18.

### Sequence alignment and data analysis

Multiple alignment and analysis of ITS DNA sequences were carried out using Clustal X2 [30]. Genetic distances were computationally analyzed using MEGA, version 4.0, according to the Kimura Two Parameter (K2P) model [31]. The neighbor joining (NJ) consensus tree was also constructed using MEGA 4.0.

### Mice

Seven to eight-week-old male C57BL/6 mice (17–20 g) were purchased from the National Laboratory Animal Center (Tainan, Taiwan). Every five mice were fostered in one cage in a special pathogen free (SPF) animal room kept at 22°C with 55% relative humidity on a 14-h light/10-h dark cycle. The mice were fed with a sterilized diet (Laboratory Autoclavable Rodent Diet 5010, USA) during their one-week acclimatization.

### Dextran sodium sulfate-induced acute murine colitis model

After acclimatization, test mice were randomized into four groups: vehicle control, dextran sodium sulfate (DSS, colitis control), sulfasalazine (positive control, Sigma-Aldrich, Lot No. 460842) and the treatment group (n = 10). All mice received normal sterilized drinking water from day -7 to 0. Subsequently, acute colitis was induced by adding 2% (w/v) DSS (molecular weight: 36,000–50,000 Da, MP Biochemicals, Solon) solution to the drinking water for each group (except the vehicle control) from day 0 to day 8 as described previously [10]. The DSS solution was filtered and changed every three days and mean DSS consumption was recorded. Throughout the whole experimental period (i.e., from 7 days before DSS treatment to 8 days post DSS treatment, with a total of 15 day treatment duration), mice in the control and DSS groups were orally administrated with vehicle control solution (sterilized water as the vehicle). Mice in the treatment group were fed by oral gavage with water extracts derived from various *Wedelia* species dissolved in sterilized Milli-Q water at a dose of 50 mg/kg body weight. The dose of 50 mg/kg as the optimal dosage is based on our preliminary test and on our results previously published in PLOS ONE [9]. Sulfasalazine was orally administered as a positive control at a dose of 200 mg/kg after the sterilized drinking water was changed to a 2% DSS. Sulfasalazine solution was dissolved in 0.5% cellulose, the 0.5% cellulose was first dissolved in saline and autoclaved, and then Tween 80 was added to 1%. At the end of the experiment, all test mice were weighed and sacrificed by cervical dislocation.

### Evaluation of colitis severity

To assess the extent of colitis in test mice, body weight, stool consistency and fecal occult blood were monitored daily using a method modified from Cooper et al. [32]. Hemoccult was assessed by the guaiac paper test (Hemoccult SENSA, Beckman Coulter) and scored as follows: 0, normal; 2, trace positive; 3, strong positive; 4, gross bleeding [33–35]. The definitions of the disease activity index (DAI) are outlined in S1 Table. At the end of each experiment, all mice were weighed then sacrificed by cervical dislocation. The large intestines were excised, colon length was measured and washed with 0.9% saline solution after removing the cecum and placed into 10% neutral-buffered formalin for fixation.

### Histological evaluation of colitis severity and hematoxylin-eosin staining

Excised fresh colons were cut into defined 0.5 cm long segments and fixed in 10% formalin overnight. The samples were then dehydrated by passing through a gradual ethanol series (from 70% to 100%) and dimethylbenzene, and then embedded in paraffin. Tissues were sectioned to 4 μM thicknesses using a microtome, and slides were prepared. The paraffin was removed by passing samples through dimethylbenzene and a gradual ethanol series (from 100% to 95%). Tissue slides were then stained with hematoxylin and eosin (Muto pure chemicals, Lot. No 3008–1). Finally, tissue sections were mounted in Histokitt mounting medium (Assistant) for microscopic examinations.

### High performance liquid chromatography

For analysis by high performance liquid chromatography (HPLC), powders of plant tissue extracts were dissolved in deionized water at a concentration of 4 mg/ml, and centrifuged at 14,000 rpm for 10 minutes to remove debris. Test extracts (4 mg/ml, 20 μl) were analyzed by HPLC (Agilent 1100 series with a G131A Quat pump) using a C_18-_AR-II 5 μm column (250 × 4.6 mm Cosmosil) at a flow rate of 1 ml/min with a elution gradient program of 15% methanol with 0.05% trifluoroacetic acid (TFA) to 70% methanol with 0.05% TFA over 30 minutes, and then held at 100% methanol with 0.05% TFA for 20 min. A photodiode array detector (DAD, Agilent 1100) was used to identify various plant metabolites at 254 nm, 280 nm, 300 nm and 350 nm.

### LC-ESI-Q-TOF MS analysis

For LC-MS analysis, acetonitrile (ACN) with 0.1% formic acid (FA) and water with 0.1% formic acid (LC-MS grade, J. T. Baker, Phillipsburg, NJ) were used in the mobile phase. Sulfadimethoxine and formic acid were purchased from Fluka (Germany). LC-MS was performed with a LC system, (Acquity UPLC, Waters, Millford, MA), coupled to a hybrid Q-TOF mass spectrometer (Synapt HDMS, Waters, Manchester, UK). Test samples were separated online with a reverse-phase column, (HSS T3 C18, 1.8 μm, 2.1 mm × 150 mm, Waters, Milford, MA), which was kept in a column oven at 40°C. The mobile phases for positive ion mode consisted of 0.1% formic acid in 2% ACN (buffer A) and 0.1% formic acid in 100% ACN (buffer B). The mobile phase in the negative-ion mode consisted of 2% ACN (buffer A) and pure 100% AcCN (buffer B). The injection volume was 10 μL and the mobile phase flow rate was 400 μl/min using a 4 min gradient of 5–95% acetonitrile/water. The mass spectrometer equipped with a lock electrospray ionization probe was operated in both positive and negative mode. The electrospray voltage was set to 3 kV for the positive ion mode and -2.5kV for the negative ion mode, and the cone voltage was 40 V. The cone and desolvation gas flow were 50 and 700 μL/h, respectively. A lock mass calibration of sulfadimethoxine (0.5 mg/L) in water/MeOH (50:50 v/v) was introduced by the HPLC pump (LC-10ATVP, Shimadzu, Japan) and split to the lockspray probe at 5 μL/min. The acquisition method was set to one full MS scan (50–990 m/z) with a 0.2 sec scan time in centroid data mode.

### Processing of data files

The LC-MS data were analyzed by MarkerLynx XS version 4.1 SCN639 software (Waters, Milford, MA). In this application, the 0.75–5.75 min LC-MS data were peak-detected and noise-reduced in both the LC and MS domains. The extracted peak information was also processed to remove the peak-to-peak noise, deisotope and filter the MS peaks with an intensity lower than 50 counts. A list of the intensities of the processed peaks for each of the LC-MS data was generated using retention time (RT) and m/z data pairs as the identifier of each peak. The processed data for each of the samples were combined and aligned for each of the RT-m/z pair to generate the final data table. The ion intensities for each peak were then normalized within each sample and the 3-dimensional data, peak identifier (RT-m/z pair), sample name, and ion intensity were analyzed by principle component analysis (PCA).

### Statistical analysis

Results (including body weight, disease activity index, colon length, histological score) are presented as mean ± standard deviation (SD). Overall differences between test groups were determined by ANOVA, followed by Dunnett’s test. A P value of less than 0.05 was considered significant.

### Ethics statement

All animal studies for this research work were performed in strict accordance with the recommendations in the Guide for the Institutional Animal Care and Use Committee (IACUC) of Academia Sinica. The protocol was approved by the IACUC of Academia Sinica (Protocol ID: 11-10-232) (S1 File). This research complies with the Animal Research: Reporting of *In Vivo* Experiments (ARRIVE) guidelines (S1 ARRIVE Checklist). All *Wedelia* plant species are not the endangered or protected species, and were not collected from national park or protected area. No specific permissions were required for collection of plants from these locations/activities.

## Results

### Macroscopic characterization and identification of *Wedelia* species

**Fig 1:**
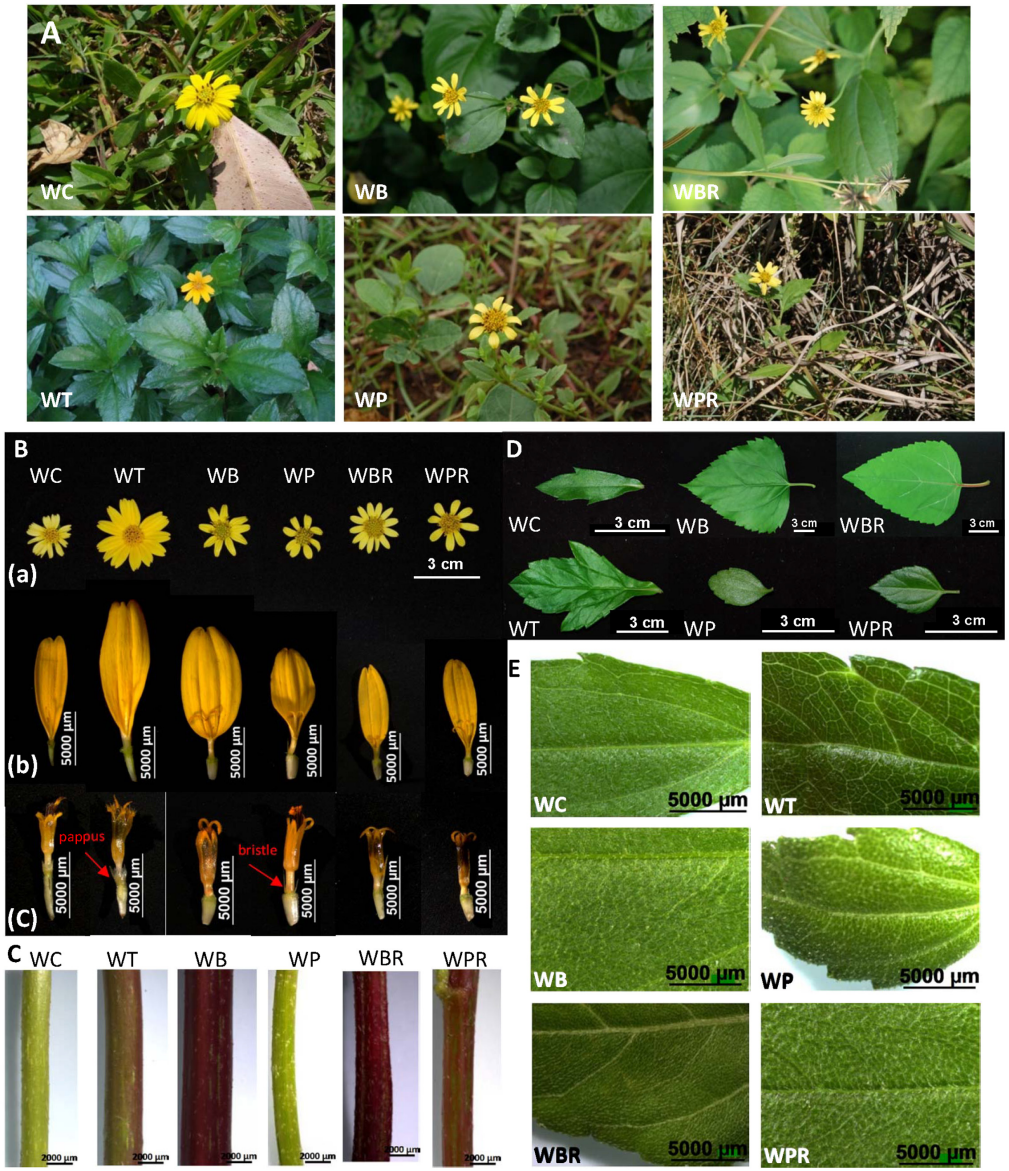
Macroscopic characteristics of *Wedelia* species. **A,** habitat; **B**, flower structures: (a) flowers, (b) ray florets, (c) disc florets; **C,** stems; **D&E,** leaves.

Four species and two additional varieties of *Wedelia* grow naturally in Taiwan (Table 2) [22]. These *Wedelia* species or cultivar strains were collected from areas in the northern and central counties of Taiwan (details in Table 1). Some of these *Wedelia* species are similar in morphological appearance (Fig 1A); and all bear yellow flowers (Fig 1B). The number of ray florets varies: *W*. *trilobata* has 8 to 14; *W*. *chinensis* and *W*. *biflora* have 8 to 12; *W*. *prostrata* and *W*. *prostrata* var. *robusta* have 8; and *W*. *biflora* var. *ryukyuensis* having 13 to 15 (Table 3). Ovaries of *W*. *chinensis* and *W*. *trilobata* are crowned with a cup-shaped pappus on the summit. *W*. *biflora*, *W*. *prostrata*, *W*. *prostrata* var. *robusta* and *W*. *biflora* var. *ryukyuensis*, on the other hand, do not have a pappus or it has degenerated into one or two bristles (Fig 1B(b) and 1B(c)). The shape of the ovaries of *W*. *chinensis* is similar to those of *W*. *trilobata*. And *W*. *biflora*, *W*. *prostrata*, *W*. *biflora* var. *ryukyuensis* and *W*. *prostrata* var. *robusta* have remarkably similarly shaped ovaries (Fig 1B(b) and 1B(c)) that differ from those of *W*. *chinensis* and *W*. *trilobata*.

**Table 2:** *Wedelia* species in Taiwan.

Species	Acronym	Additional Varieties	Acronym
*Wedelia chinensis*	WC		
*Wedelia trilobata*	WT		
*Wedelia biflora*	WB	*Wedelia biflora* var. *ryukyuensis*	WBR
*Wedelia prostrate*	WP	*Wedelia prostrata* var. *robusta*	WPR

**Table 3:** Summary of macroscopic and microscopic characteristics observed for stem and leaf tissues of *Wedelia* species.

	Species					
Characteristics	WC	WT	WB	WP	WBR	WPR
Hair type on leaf surface	appressed pilose	pubescent	appressed strigose	strigose	appressed strigose	strigose
Leaf shape	linear-oblong	trilobate	ovate-lanceolate	oblong	ovate-lanceolate	ovate
Pappus on ovary summit	+	+	1–2 bristles	1–2 bristles	1–2 bristles	1–2 bristles
Number of ray florets	8–12	8–14	8–12	8	13–15	8
Stem shape	circular	polygonal	cross-shape	cross-shape	cross-shape	cross-shape
Number of vascular bundles in stem	12–17	17–22	20–29	12–26	18–28	20–29
Resin ducts in cortex	+	+	+	+	+	+
Resin ducts in pith	+	+	+	-	+	+
Resin ducts in leaves	+	+	+	+	+	+

Abbreviations, including WC, WT, WB, WP, WBR and WPR, are described as shown in Table 2.

Stems of *W*. *biflora*, *W*. *prostrata*, *W*. *biflora* var. *ryukyuensis* and *W*. *prostrata* var. *robusta* exhibit deep angular structures with strigose hairs (Fig 1C). In contrast, the stem of *W*. *trilobata* is polygonal or circular in shape with pubescent hairs, and that of *W*. *chinensis* is circular with appressed-pilose hairs (Fig 1C). The *W*. *chinensis* leaf is linear-oblong with appressed-pilose hairs; the *W*. *trilobata* leaf is always three-lobate with pubescent hairs; *W*. *biflora* and *W*. *biflora* var. *ryukyuensis* leaves are ovate-lanceolate with appressed-strigose hairs; *W*. *prostrata* is oblong with densely strigose hairs; and *W*. *prostrata* var. *robusta* is ovate with appressed-strigose hairs (Fig 1D and 1E, Table 3).

### Anatomical analysis of plant tissues

**Fig 2:**
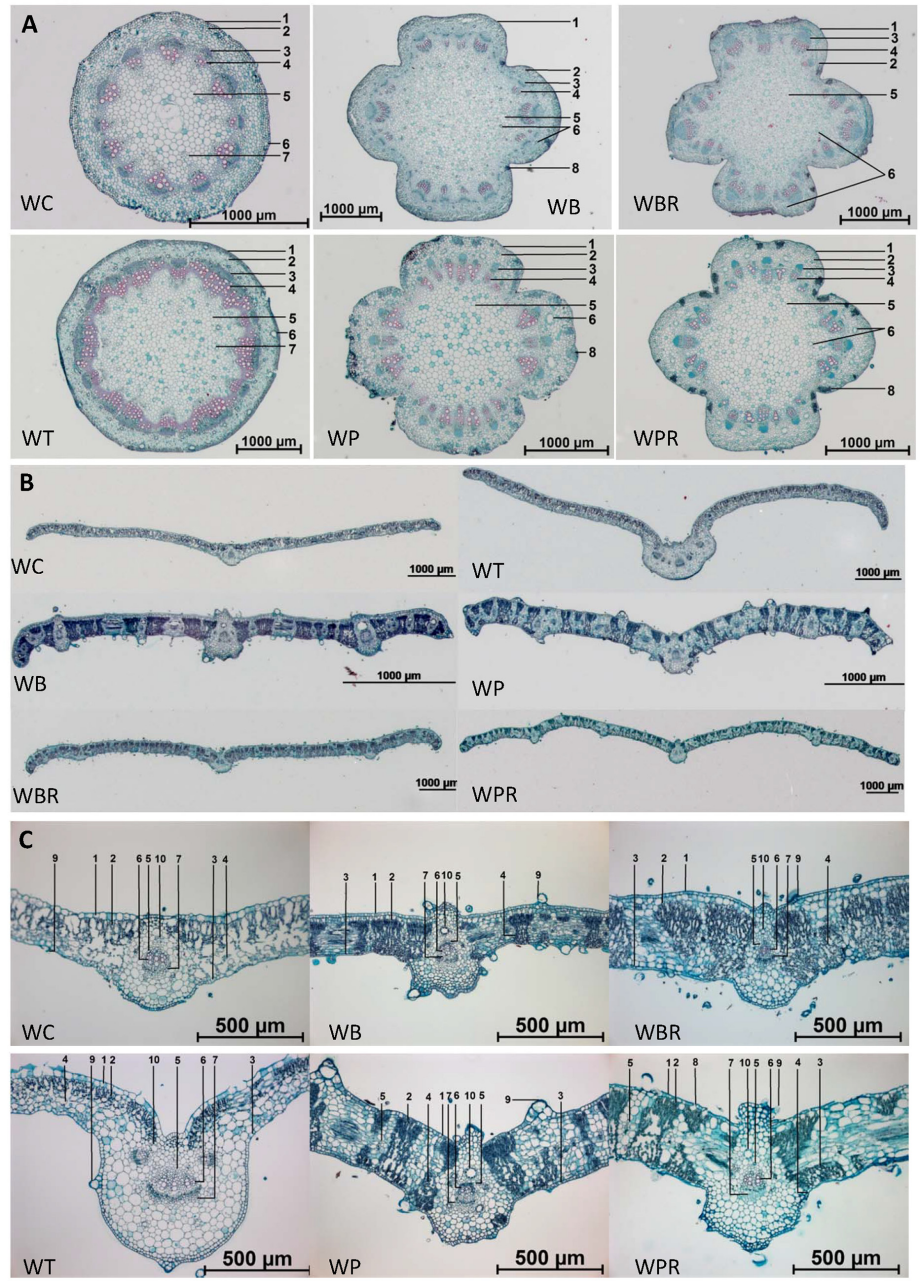
Transverse sections of stems and leaves of *Wedelia* species. **A,** Tissue sections of stems: 1. Epidermis; 2. Cortex; 3. Phloem; 4. Xylem; 5. Pith; 6. Resin duct; 7. Pro-resin duct; and 8. Secretory cells. **B & C,** Tissue sections of leaves: 1. Upper epidermis; 2. Palisade tissue; 3. Lower epidermis; 4. Spongy tissue; 5. Bundle sheath; 6. Xylem; 7. Phloem; 8. Stoma; 9. Trichome; 10. Resin duct.

The transverse tissue section of the stem of *W*. *chinensis* is circular in outline and that of *W*. *trilobata* is virtually polygonal with the vascular bundles cross-connected; those of *W*. *biflora*, *W*. *prostrata*, *W*. *prostrata* var. *robusta* and *W*. *biflora* var. *ryukyuensis* are cross-shaped (Fig 2A). According to observations of free-hand cross sections (data not shown), *W*. *chinensis* has 12 to 17 vascular bundles in the stems; *W*. *trilobata* has 17 to 22; *W*. *biflora* has 20 to 29; *W*. *prostrata* has 12 to 26; *W*. *prostrata* var. *robusta* has 18 to 28; and *W*. *biflora* var. *ryukyuensis* has 20 to 29 (Table 3). With the exception of *W*. *prostrate*, all of the *Wedelia* species examined had resin ducts which are located in the cortex and pith of stems, (S1 Fig and Table 3). The resin ducts of *W*. *prostrata* are only located in the cortex (S1 Fig). In addition to resin ducts, secretory cells can be found in the stems of *W*. *biflora*, *W*. *prostrata*, *W*. *prostrata* var. *robusta* and *W*. *biflora* var. *ryukyuensis*. The arrangements of the vascular bundles in these *Wedelia* stems can vary among the species (S1 Fig).

The transverse sections of leaves of *W*. *chinensis* and *W*. *trilobata* are quite similar and their leaves are generally thinner than those of the other species (Fig 2B and 2C). *Wedelia trilobata* usually has multiple vascular bundles in the midrib whereas the other *Wedelia* species have a single vascular bundle (Fig 2B and S2 Fig). This feature may be related to the fact that *W*. *trilabata* has three-lobate leaves. The arrangements of spongy and palisade tissues in *W*. *chinensis* are similar to those in *W*. *trilobata*; in *W*. *biflora* they are similar to those in *W*. *biflora* var. *ryukyuensis*; and in *W*. *prostrata* they are similar to those in *W*. *prostrata* var. *robusta* (Fig 2B and 2C). All the species and varieties have trichomes on both the upper and lower epidermis (Fig 2C and S2 Fig). They also have resin ducts, usually located near the vascular bundles in the midrib and veins (S2 Fig and Table 3).

### Comparative molecular and genotyping analyses of internal transcribed spacer sequences of *Wedelia* species

**Fig 3:**
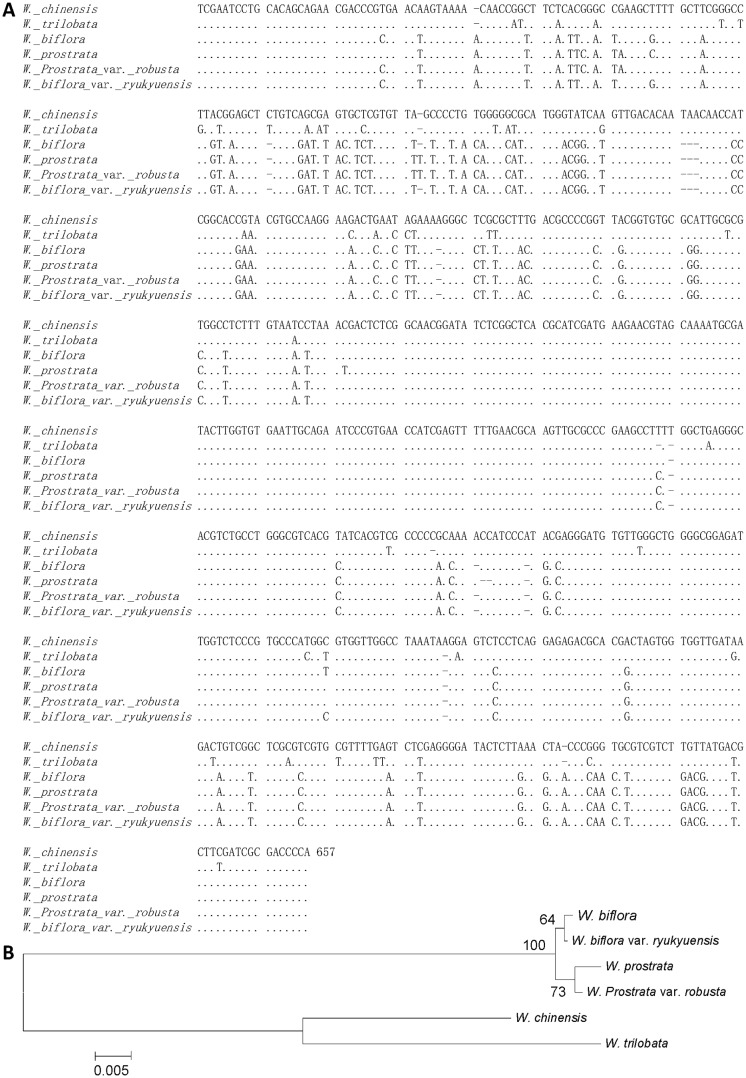
Multiple alignment of the ITS regions of *Wedelia* species in Taiwan. **A,** ITS1 and ITS2 genes are located between 1 and 256 bp, and between 422 and 657 bp of the ribosomal DNA (rDNA) gene, respectively. The 5.8S rDNA is located between 257 and 421 bp in the rDNA gene. **B,** The neighbor-joining consensus tree of *Wedelia* species in Taiwan established based on the ITS sequence analogy (Bootstrap: 500 replicates, seed: 954).

The internal transcribed spacer (ITS) regions of analyzed *Wedelia* species were measured to be between 637 and 646 bp (Table 4) in length. The ITS sequences of *W*. *biflora* (accession number JF934953), *W*. *biflora* var. *ryukyuensis* (accession number JF430396) and *W*. *prostrata var*. *robusta* (accession number JF430395) have been submitted to GenBank. And the ITS sequences of *W*. *chinensis*, *W*. *trilobata* and *W*. *prostrate* are the same as the sequences AY303442 (*W*. *trilobata*), AY947415 (*W*. *chinensis*) and AY947412 (*W*. *prostrate*) submitted previously. They are composed of ITS1, 253 to 256 bp; 5.8S, 162 to 167 bp; and ITS2, 217 to 228 bp (Table 4). The GC content of the ITS in all species was determined to be approximately 53.2% in ITS1, 53.4% in 5.8S, and 57.5% in ITS2. We detected 124 variable sites in the ITS region, and among them 76 variable sites were detected in ITS1, five in 5.8S and 43 in ITS2 (Fig 3A). The smallest genetic distance was found at 0.002 between *W*. *biflora* and *W*. *biflora* var. *ryukyuensis* and the largest genetic distance was 0.161 between *W*. *prostrata* and *W*. *trilobata* (Table 5). According to the neighbor-joining tree analysis, the tested *Wedelia* can be divided into two clades: one clade composed of *W*. *prostrata*, *W*. *biflora*, *W*. *prostrata* var. *robusta* and *W*. *biflora* var. *ryukyuensis*, and a second clade composed of *W*. *chinensis* and *W*. *trilobata* (Fig 3B). This neighbor-joining tree was found to appropriately correspond not only with the histological analysis of stem and leaf tissues of these *Wedelia* species but also with the HPLC chemical profiles of the water-extracted metabolites present in these *Wedelia* species (see below).

**Table 4:** Nucleotide sequence length of ITS1, 5.8S rRNA and ITS2 genomic DNA fragments of four species and two additional varieties of *Wedelia*.

Species	ITS regions (bp)				Accession number
	ITS1	5.8S	ITS2	Total	NCBI database
*W*. *chinensis*	254	164	228	646	AY947415*^1^
*W*. *trilobata*	256	162	218	636	AY303442*^2^
*W*. *biflora*	253	163	225	641	JF934953
*W*. *prostrata*	253	167	217	637	AY947412*^1^
*W*. *biflora* var. *ryukyuensis*	253	163	225	641	JF430396
*Wedelia prostrata* var. *robusta*	253	167	218	638	JF430395

*^1^ The sequences AY947415 and AY947412 were submitted to GenBank by Yuan, C.I., Hsieh, Y.C. and Chiang, M.Y.

*^2^ The sequence AY303442 was submitted to GenBank by Dias de Moraes, M., Panero, J.L. and Semir, J.

**Table 5:** Kimura’s two-parameter’s genetic distances as determined based on total ITS sequence.

Species	WC	WT	WB	WP	WPR	WBR
WC						
WT	0.071					
WB	0.146	0.155				
WP	0.148	0.161	0.009			
WPR	0.146	0.159	0.006	0.005		
WBR	0.144	0.157	0.002	0.008	0.005	

### Analyses of chemical fingerprints of *Wedelia* species by HPLC

**Fig 4:**
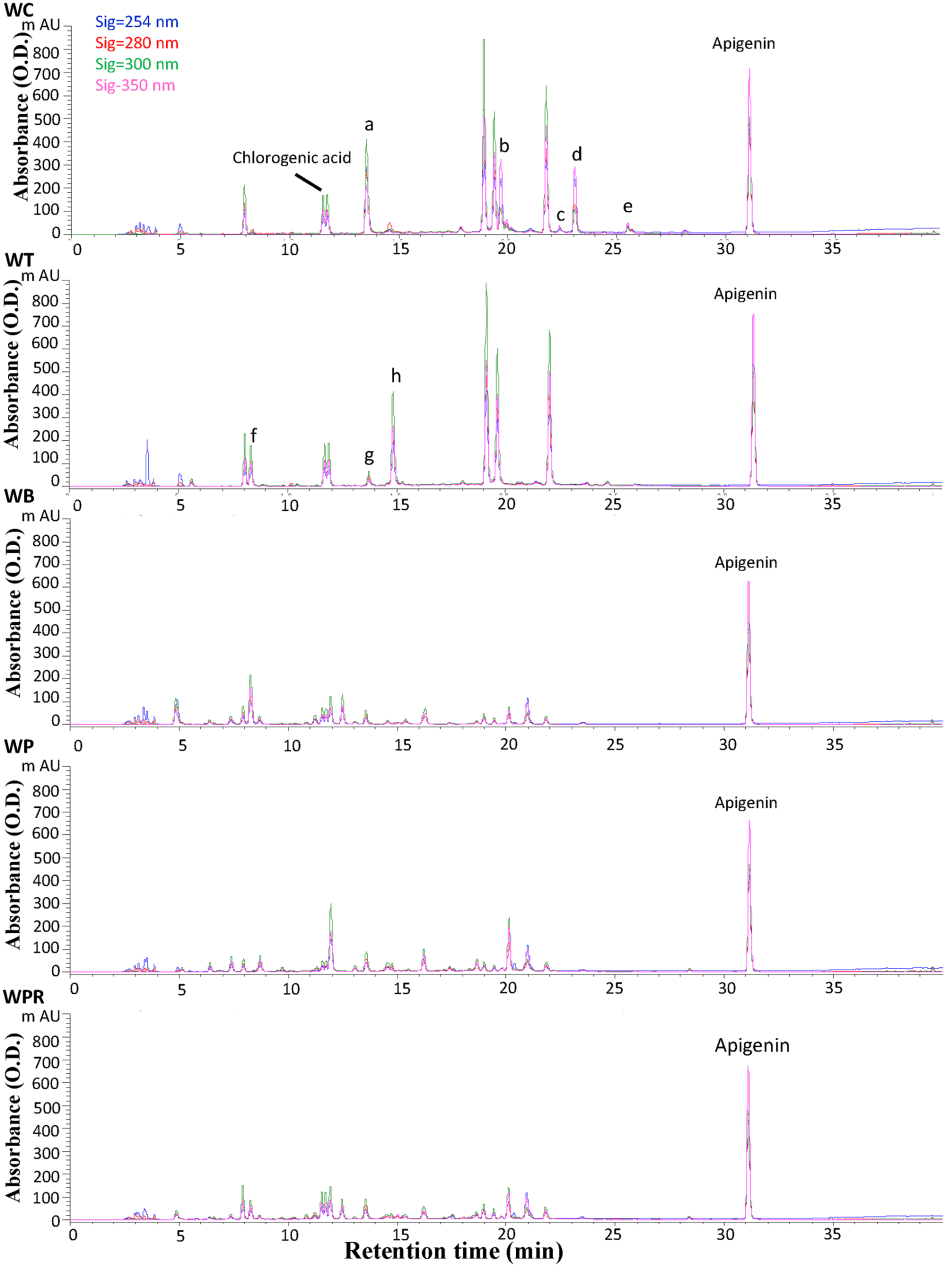
HPLC chromatograms of hot water extracts of five *Wedelia* species. Apigenin was supplemented only as an internal standard to calibrate the HPLC data. The colors of signals, blue, red, green and pink, denote different absorbance levels of 254 nm, 280 nm, 300 nm and 350 nm, respectively. The peaks of a, b, c, d, e, f and g represent the different peaks detected between WC and WT extracts.

HPLC analysis revealed five distinguishable metabolite profiles from tissue extracts of test *Wedelia* species (Fig 4). The population of *W*. *biflora* var. *ryukyuensis* plants in plantations or fields in Taiwan was found to be limited, impeding harvesting of sufficient materials for systematic studies of this species. Chromatogram peaks exhibiting specific metabolite profiles were complemented using a photo-diode array detector (S3 Fig). Thus, the metabolite peaks were characterized by both retention time and UV spectra. Some peaks exhibiting the same retention time for both *W*. *chinensis* and *W*. *trilobata* plants also have similar UV spectra (Fig 4 and S3 Fig). We observed that peaks f, g and h were present only in *W*. *trilobata*, whereas peaks a, b, c, d and e were present only in *W*. *chinensis* (Fig 4, WC and WT). Although the retention time of peak a in *W*. *chinensis* is similar to peak g in *W*. *trilobata*, their UV spectra are quite different (S3 Fig). The UV spectra for several peaks shown in S3B Fig in *W*. *chinensis* and *W*. *trilobata* are similar to that of caffeic acid, indicating they are likely caffeic acid derivatives (S3A and S3B Fig). Comparison of these two HPLC metabolite profiles with those from the other three *Wedelia* species/variants revealed that *W*. *biflora*, *W*. *prostrata* and *W*. *prostrata* var. *robusta* have relatively similar metabolite profiles as a group (Fig 4), that are dramatically different from those of *W*. *chinensis* and *W*. *trilobata*.

We further identified a number of phytochemical peaks as candidate “index compounds”, which may identify the metabolite profiles of the *Wedelia* species. We observed that the retention time and UV spectra for peaks 3 and 4 were quite similar to that exhibited by chlorogenic acid, while peak 5 was similar to ferulic acid. These results were further verified by LC-MS using authentic pure compounds for comparison (data not shown). We hence suggest that the various secondary metabolites extracted from the five tested *Wedelia* species may all contain chlorogenic acid or its analogue(s). Another compound, ferulic acid, may also be an index compound for both *W*. *chinensis* and *W*. *trilobata* extracts. We and others [36–38] have reported a spectrum of bio-activities of chlorogenic acid, caffeic acid and ferilic acid on various mammalian systems. On the other hand, these phytochemicals, as secondary metabolites, were also shown to confer important stress or pathogen-responsive activities in plants. Possible correlation and significance of these cross-kingdom bio-activities are addressed in Discussion.

### UPLC-ESI-Q-TOF MS analyses of chromatograph fingerprints of *Wedelia* species

**Fig 5:**
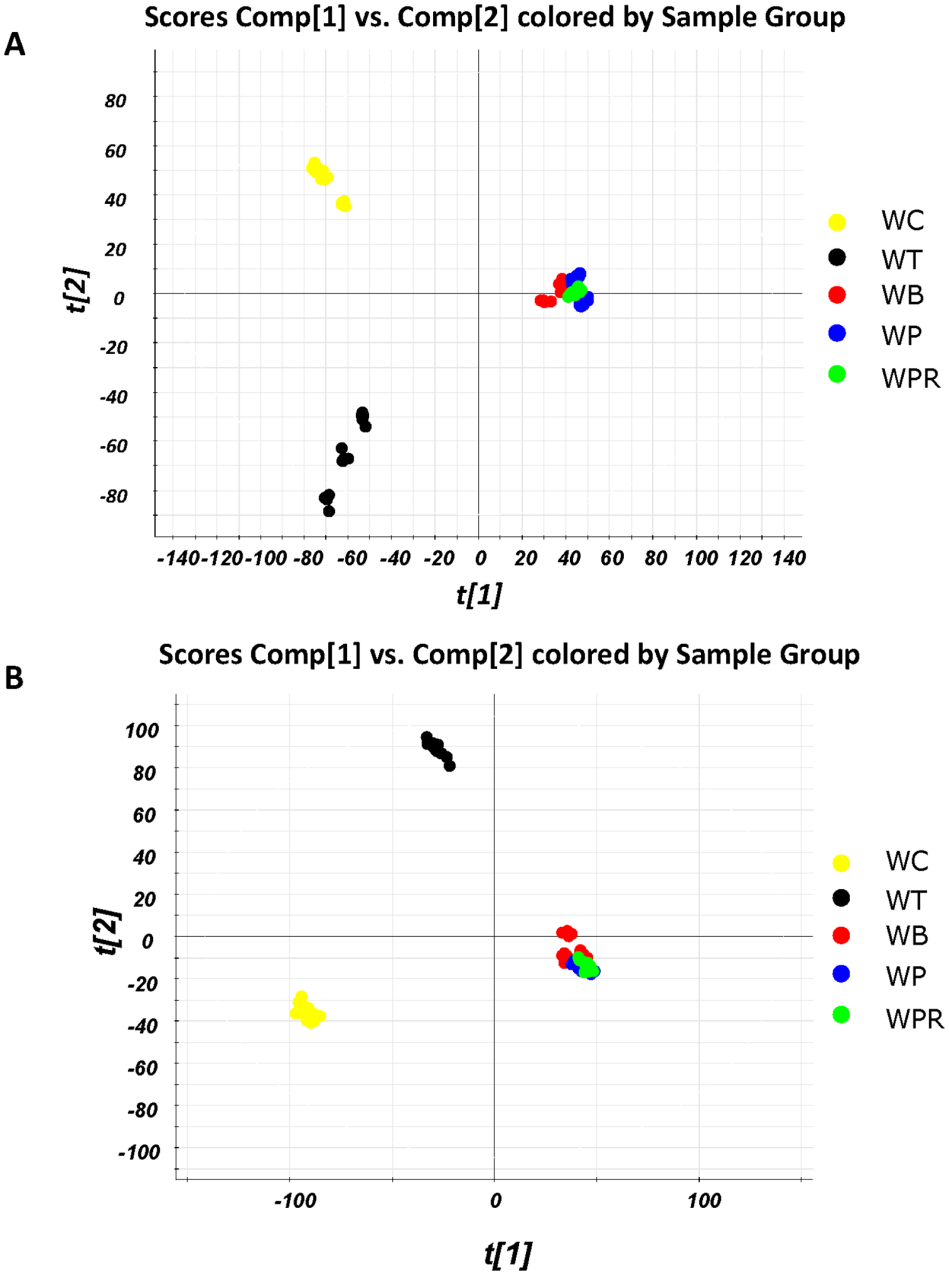
PCA analysis of plant metabolites from aqueous tissue extracts of *Wedelia* species using UPLC-ESI-MS. **A,** Electrospray ionization positive (ES^+^) LC-MS data. **B,** Electrospray ionization negative (ES^-^) LC-MS data.

To further analyze the chemical constituents of plant metabolites from test *Wedelia* species, we investigated the metabolite profiles using the UPLC-ESI-Q-TOF MS system. Various chemical components were detected by this MS system in both ES^+^ and ES^-^ modes (S4 Fig) suggesting that the sensitivity of MS we set to optimize the assay conditions was adequate for the present study. Using the LC-MS dataset, we obtained 3164 peaks and 1849 peaks from the five *Wedelia* species in the ES^+^ and ES^-^ mode, respectively. Subsequently, principal component analysis (PCA) was performed to analyze these peaks. PCA for data from both ES^-^ and ES^+^ modes revealed a clear variation in the metabolite content, effectively separating the five *Wedelia* species into the following three groups: (1) *W*. *chinensis*; (2) *W*. *trilobata*; and (3) *W*. *biflora*, *W*. *prostrata* and *W*. *prostrata* var. *rubosta* (Fig 5).

### Functional analysis of *in vivo* anti-inflammatory bioactivities in DSS-induced acute colitis mice

**Fig 6:**
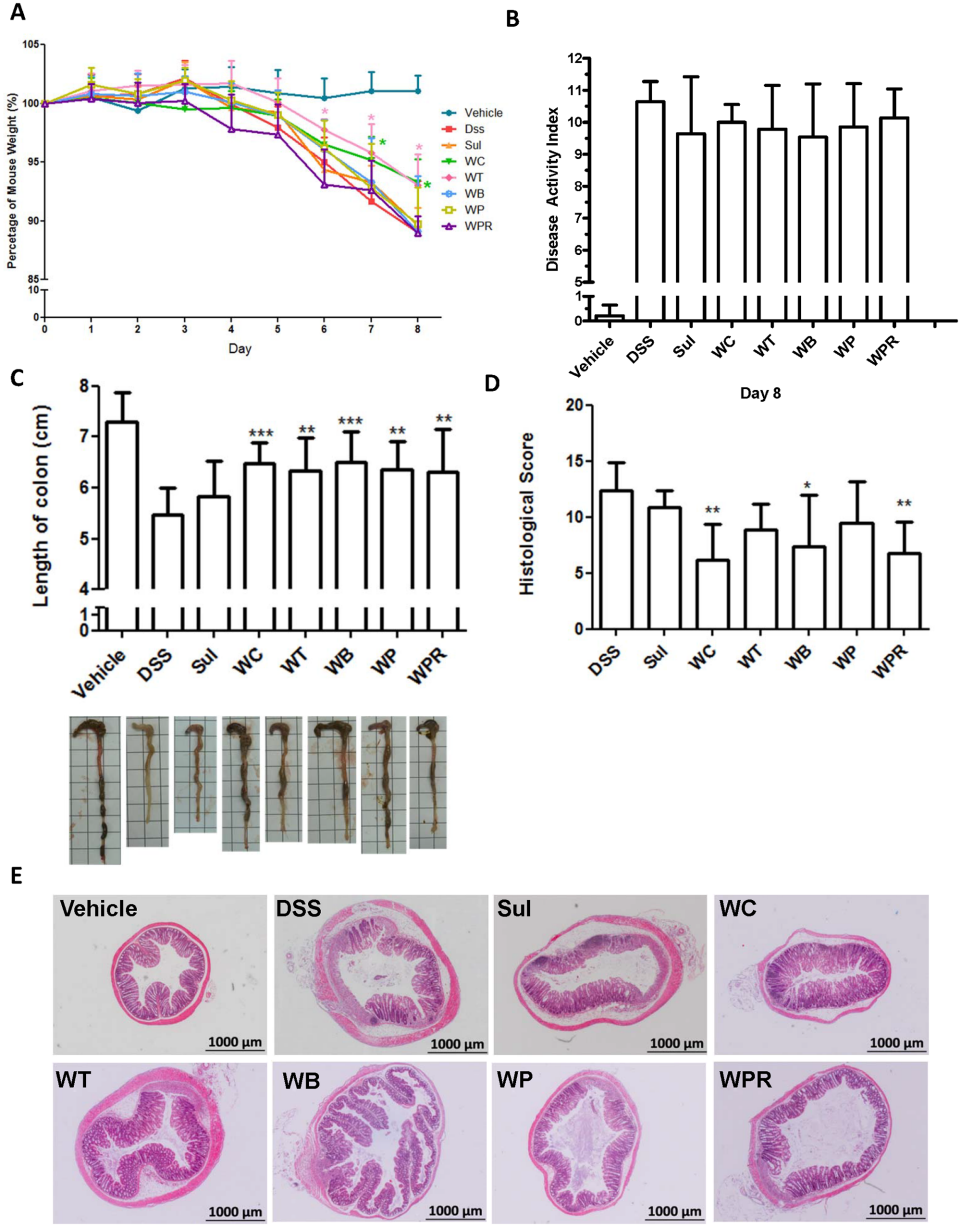
Effect of different extracts of *Wedelia* species in mice with DSS-induced acute colitis. Mice were simultaneously administered orally with vehicle (sterilized water), Sul: sulfasalazine (positive control, 50 mg/kg) or different extracts of *Wedelia* species (50 mg/kg), respectively. **A,** Percentage of murine body weight. **B,** Disease activity index. **C,** Colon length. **D,** Histological score. **E,** Histopathology of mice with DSS-induced acute colitis. Data are expressed as mean ± SD (n = 10). WC and WT, respectively *P < 0.05, **P < 0.01, ***P < 0.001, significant difference compared with the DSS group.

To determine the possible specific anti-inflammatory functions of *Wedelia* species, we used a DSS-induced acute murine colitis model to evaluate the bioactivities of various *Wedelia* herbal extracts. Throughout the experiment, we monitored the oral intake of drinking water spiked with 2.0% DSS: the consumption level of DSS-spiked water was found to be statistically indistinguishable among all test groups (data not shown). Sulfasalazine, a commercialized drug for treating and preventing relapse of IBD was used as the positive control for this experiment [39,40]. Under normal conditions, i.e., DSS was not added to drinking water, *Wedelia* herbal extracts did not promote mouse body weight loss (data not shown). Mice treated with 2% DSS in drinking water developed typical symptoms of acute colitis including diarrhea, rectal bleeding and loss of body weight. Comparison of the DSS group with the vehicle group showed that the relative body weight (%) of the DSS-treated group decreased significantly on days 6, 7 and 8 (Fig 6A). After treatment with 2% DSS, mouse body weights were found to be significantly lower in the DSS, sulfasalazine (Sul), WB, WP and WPR-treated groups than in the WC and WT groups on days 7 and 8 (Fig 6A). The disease activity index (DAI) was calculated according to the severity of clinical colitis symptoms (S1 Table). Statistical analysis revealed that the DAI scores for various *Wedelia* species groups were not significantly different from that for the DSS group (Fig 6B). Although we can not co-relate clearly the bioactivities of different *Wedelia* plant extracts to the DAI score, we consider that the specific difference between the WC and DSS only group is in agreement with other data shown in Fig 6 and as we previously reported [9]. Decrease in colon length is widely accepted as a critical symptomatic parameter in DSS-induced colitis. Fig 6C shows that the colon length of test mice was significantly decreased after eight days of DSS administration. A comparison of the *Wedelia* extract-treated experimental groups and the untreated, DSS only group showed that WC, WT, WB, WP and WPR treatments effectively protected the colon tissues of test mice from physically contracting or shortening (Fig 6C). These treatments, except for WT and WP, also attenuated the histopathological manifestations in test colons (Fig 6D and 6E). Microscopic examination of haematoxylin and eosin (H&E)-stained middle colon tissue sections from groups administered 2% DSS alone for eight days showed a substantial decrease in colon length, regeneration or losing of the large area crypts, disruption of epithelial lining, massive infiltration of the lamina propria and submucosa tissues by inflammatory cells, and submucosal erosion with edema (Fig 6E). Comparison of colon tissue sections from various *Wedelia* extract-treated and DSS only-treated mice showed a notably lower degree of edema, crypt abscesses, and erosion with infiltration of mononuclear cells or other lymphocytes into the mucosal tissues in the WC-treated groups as compared to the DSS only-treated group. Interestingly, the WP-treated group showed little or no reduction in colitis symptoms on histopathological examination, whereas the WC group showed histological features, tissue size and morphology that closely resembled those of vehicle (control) mice (Fig 6E). Sulfasalazine-treatment had a modest effect on colon length (Fig 6C), histological score (Fig 6D) and histopathology microscopy (Fig 6E). The histological scores, as quantitatively determined according to standard histopathological examinations (S2 Table), were also drastically decreased in the WC-treated mice, with varying degrees of reduction in the WPR and WB groups (Fig 6D). These results corresponded reasonably well with the histopathological examinations. Together, the multiple assay results obtained from the DSS-induced acute colitis murine model (Fig 6) suggest that dietary treatment with WC can markedly ameliorate both the colitis-like symptoms and the tissue damage caused by DSS-induced inflammation and tissue-wounding in colons of DSS-induced acute colitis mice.

## Discussion

In this study, we used a combined technological approach to authenticate, validate or profile the various species/varieties and biodiversities of *Wedelia* plants that grow naturally in Taiwan, and further investigated their reputed bioactivities *in vivo* in a clinically-relevant animal/disease model as an example of a systematic exploration of plant secondary metabolites found in traditional medicinal plants. Among various medicinal plants from the *Wedelia* genus commonly found in Taiwan, as compared to the untreated (DSS-treated only) mice, only the phytoextract from the *W*. *chinensis* species exhibited statistically significant anti-inflammatory bioactivity on DSS-induced murine colitis. This result may be connected to two groups of secondary plant metabolites, caffeic acid derivatives and chlorogenic acid, present in *W*. *chinensis* extracts. Further study is needed however to verify whether these index compound-like phytochemicals are indeed the active ingredient(s) of the detected bioactivities.

Caffeic acid and derivatives are efficacious phytochemicals that may help defend plants against abiotic and/or biotic stresses. They were reported to help protect plants against plant pathogens, including fungi, bacteria and insects [41–43]. They have also been shown to contribute or be associated with the control of physical or chemical stresses including drought, salinity and heat [44–46]. On the other hand, the anti-oxidant, anti-carcinogenic, anti-inflammatory, immunomodulatory and even anti-colitis activities of caffeic acid and some of its derivatives have also been previously reported by our own group and others [36,47–52]. Chlorogenic acid has also been reported to exhibit various bioactivities in mammalian systems, including anti-inflammatory and cytokine-modulatory activities [53–59]. Caffeic acid has been recently shown to increase the expression of CYP4B1 mRNA in DSS-treated mice and effectively inhibited DSS-induced murine colitis [60]. In light of these findings, on chlorogenic acid and caffeic acid, we suggest that these two plant secondary metabolites or/and their derivatives may act target in plant extract mixtures on effective inhibition of colon inflammation in DSS-treated mice with colitis. Dietary uptake of *W*. *chinensis* plant extracts may thus confer phytomedicinal or nutraceutical effects on IBD in humans. Obviously, such claims would need to be evaluated and verified by human clinical studies or trials.

The other five species/variants other than *W*. *chinensis* tested in this study had a moderate effect on ameliorating body weight loss, colon length shortening, histological scores, DAI and histopathological activities in colitis mice. Among the various parameters evaluated (Fig 6A to 6E), *W*. *chinensis* plant extract was found to exhibit coordinated, correspondent, and routine efficacy in test bioactivities. Some plants, e.g., *W*. *prostrate*, showed little or no anti-inflammatory effects in DSS colitis model mice. Our studies hence effectively demonstrated the need for proper clarification and verification of reputed medicinal herbs for use and future development of medicinal food or botanical drugs.

Assessment of whole, fragmented, and even dried-botanical materials through microscopic identification and comparative analysis improves our capability to identify or verify medicinal plants [61]. Effective authentication of medicinal plants for botanical drug development is indeed a key requirement for future advancement of phytomedicines. In this study, in terms of biodiversity studies, we also revealed interesting and important results on the taxonomy of *Wedelia* species, an important traditional medicine herb. We integrated four different methods (as seen in Figs 1, 2, 3, 4 and 5) into a multi-parameter comparative evaluation system to identify and classify the six related *Wedelia* species/variants. According to morphological features (Fig 1), microscopic characteristics (Fig 2) and the genetic distance data we obtained via sequence analysis of ITS1 and ITS2 (Fig 3), we suggest that *W*. *prostrata* var. *robusta* could have evolved most likely as a hybrid from a cross between *W*. *prostrata* and *W*. *biflora*. This possibility was previously indicated by Peng [22]. The genetic distances we detected, i.e., 0.05 between *W*. *prostrata* var. *robusta* and *W*. *prostrata*, and 0.006 between *W*. *prostrata* var. *robusta* and *W*. *biflora*, strongly support this conclusion (Table 5).

For another comparison in grouping, *W*. *biflora* var. *ryukyuensis* is notably similar to *W*. *biflora*, not only in terms of general morphology, but also in the anatomical structure of the stems and leaves. Using macroscopic identification, we found that there was only one way to distinguish them: *W*. *biflora* var. *ryukyuensis* has a higher number of ray florets and disc florets than *W*. *biflora*, the former has 14 to15 ray florets and 45 to 70 disc florets, whereas *W*. *biflora* has 8 to 12 and 20 to 35, ray florels and disc florets respectively. It was previously reported that *W*. *biflora* var. *ryukyuensis* is a triploid plant with 2n = 45, whereas *W*. *biflora* is a diploid plant with 2n = 30 [22]. In addition to the morphological and anatomical characteristics, the genetic distance between *W*. *biflora* and *W*. *biflora* var. *ryukyuensis* was determined as 0.002 (Table 5). These results hence support the notion that *W*. *biflora* var. *ryukyuensis* is indeed likely a variety of *W*. *biflora*.

Based on the results of the neighbor-joining tree that we established using comparative sequence analysis of the ITS regions, we suggest here that these *Wedelia* species can be effectively classified into two clades (Fig 3), a result that correlates well with the similarity in the microscopic characteristics. This result on confirmation of previous speciation study also revealed the usefulness and importance of our current crosstalk (see below too) with different biotechnology study systems. Anatomical studies showed that numerous resin ducts are present in the stems and leaves of *Wedelia* species (S1 Fig and S2 Fig). The resin duct, a secretory structure, usually contains terpenoids [62] and essential oils [63]. It has been suggested that the structure of secretory cell plastids is related to their role in monoterpene synthesis [64]. Secretory structures are present in a wide spectrum of vascular plants and play an important mechanistic and ecological role in the defense against herbivores and pathogens [65]. These lipid substances, including terpenoids and essential oils, are widespread internally in secretory structures in the Asteraceae [65]. A number of studies have indicated that the chemical constituents from *Wedelia* species extracted by organic solvents may contain various terpenoids [66–69]. Our present study may suggest that specific terpenoids may be usefully employed chemical markers of *Wedelia* species. It may be also important to investigate in the future whether specific anti-pathogen and/or anti-biotic stress plant terpenoids, can also regulate inflammatory or/and immunomodulatory activities in mammalian systems. The possibility of cross-kingdom bioactivities of phytochemicals may warrant future systematic studies.

Based on the metabolite profiles of different *Wedelia* species analyzed by HPLC, *W*. *chinensis* and *W*. *trilobata* may be categorized into a group with relatively similar chemical fingerprints, whereas *W*. *biflora*, *W*. *prostrata* and their varieties can be assigned to another fingerprint group. Interestingly, although *W*. *chinensis* and *W*. *trilobata* exhibit a similar HPLC metabolite profile, we were still able to identify a number of phytochemical peaks distinctively deleted in these two different plant extracts. UPLC-MS profiling accompanied by PCA statistical analyses [2,5,70] were employed to investigate the relatively minor but still specific differences in secondary metabolites, as categorized by the mass peaks revealed from the six test *Wedelia* species. We show that *W*. *chinensis* can, in fact, be distinctly identified from *W*. *trilobata*, suggesting that these two species do represent two different phytochemical groups. Further, *Wedelia biflora*, *W*. *prostrata* and their varieties can be clearly classified into another single phytochemical group. We find this result very interesting, suggesting that HPLC and UPLC-MS in combination with specific statistical approaches can be usefully applied in the detailed and specific chemical/metabolomic profiling of mixtures of phytochemicals and their parental phytoextracts.

## Conclusions

This study set out to establish a cross-disciplinary, integrated technology platform for systematic evaluation of the safety and efficacy of traditional medicinal plants to aid the development of new botanical drugs. As now publically defined and recognized, crude or partially purified (fractionated) medicinal plant extracts or phytochemical mixtures can be employed to develop botanical drugs in USA, Europe and Asian countries. Despite this new development, the scientific and pharmaceutical communities have made very limited progress, likely due to the lack of experimental/technical know-how and useful technology platforms for systematic research and development of new phytomedicines [71]. The approach outlined here shows that a combination of taxonomy, molecular biology and chemical fingerprinting methodologies are required to effectively and accurately authenticate and characterize medicinal plants and reveal their associated bioactive compounds. Macroscopic and microscopic identification alone may not be sufficient to distinguish a spectrum of reputed or claimed traditional medicinal plants. In addition, specific genome sequencing, secondary plant metabolite profiling and index compound analysis, and specific and representative bioactivity assays *in vivo* and *in vitro* are needed to make up a sequential and integrated system to evaluate the efficacy of traditional herbal medicines and the derived phytochemicals. Here, a “real case” plant system, in contrast to a “model plant system”, was explored using the above approaches, and *W*. *chinensis* was identified and verified as candidate medicinal plant that conferred specific anti-inflammatory effects on DSS-induced murine colitis. These results provide possible leads for future use and development of evidence-based phytomedicines or botanical drugs. Further, in terms of plant science, specific plant secondary metabolites are expected to be revealed as candidate active compounds for anti-inflammatory, anti-colitis therapies. We hypothesize that these “medicinal” secondary metabolites may also confer useful anti-stress and pro-innate immunity bioactivities in host plants, due to the orthologous molecular and cellular mechanisms seen in plants. These mechanisms may well be mirrored in mammalian systems. The possible effects of this specific class of secondary plant metabolites from *Wedelia* may hence warrant further systematic investigation in the future.

## Supporting Information

S1 ARRIVE Checklist (PDF)

S1 Fig Transverse sections of stem tissues of *Wedelia* species. The arrows show resin ducts.
(TIF)

S2 Fig Transverse sections of leaf tissues of *Wedelia* species. Ph: phloem Rd: resin duct Tc: trichome X: xylem.
(TIF)

S3 Fig **A,** UV spectra detected for caffeic acid. **B,** UV spectra detected for some putative caffeic acid derivatives in the HPLC chromatograms of *W*. *chinensis* and *W*. *trilobata*. Apigenin is spiked in as the internal standard.
(TIF)

S4 Fig ES-BPI chromatograms of plant metabolites from aqueous tissue extracts of different *Wedelia* species. **A,** Electrospray ionization positive LC-MS data. **B,** Electrospray ionization negative LC-MS data.
(TIF)

S1 File Approval letter. This is to certify that the animal protocol by the following applicant has been evaluated and approved by the Institutional Animal Care and Use Committee of Academia Sinica (AS IACUC).
(PDF)

S1 Table Disease activity index. (DOCX)

S2 Table Scoring system for histological pathology study. (DOCX)
