# Supplementary figures and images for: Integrative Approach to Analyze Biodiversity and Anti-Inflammatory Bioactivity of Wedelia Medicinal Plants
Source: PLoS One. 2015 Jun 4;10(6):e0129067. doi: 10.1371/journal.pone.0129067 (PMC4456162; doi:10.1371/journal.pone.0129067)

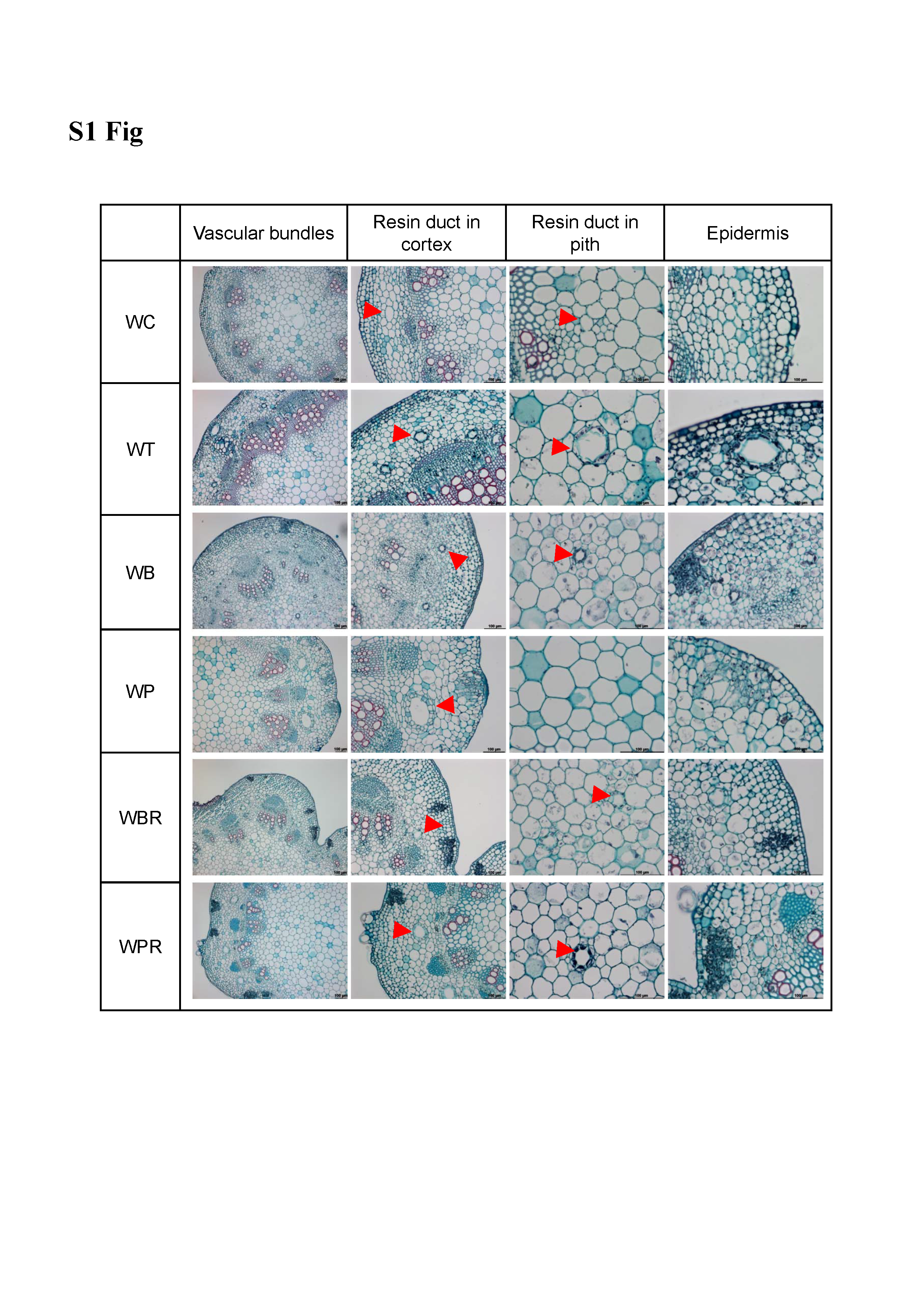

Supplement: S1 Fig — The arrows show resin ducts. (TIF) [file pone.0129067.s002.tif]

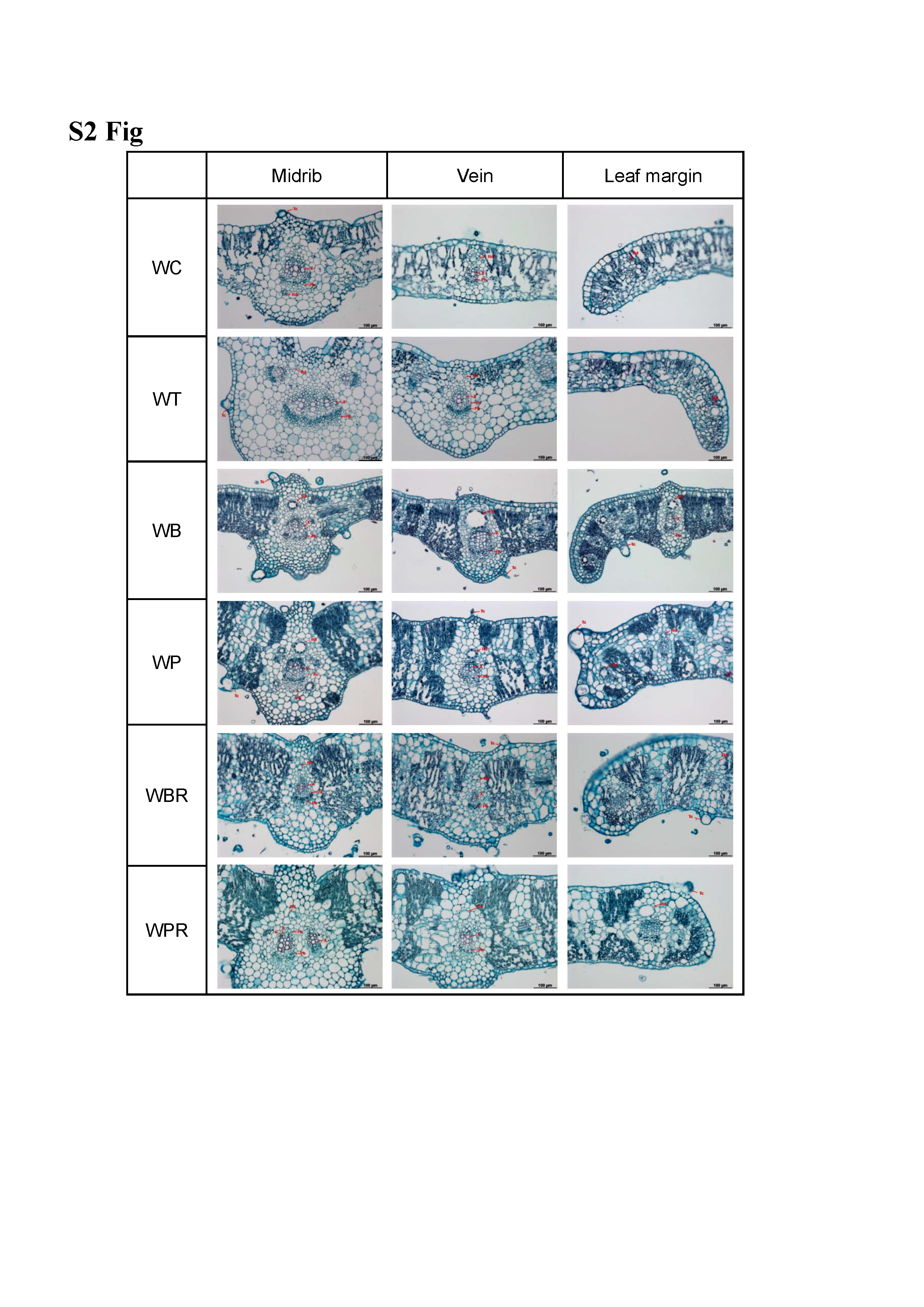

Supplement: S2 Fig — Ph: phloem Rd: resin duct Tc: trichome X: xylem. (TIF) [file pone.0129067.s003.tif]

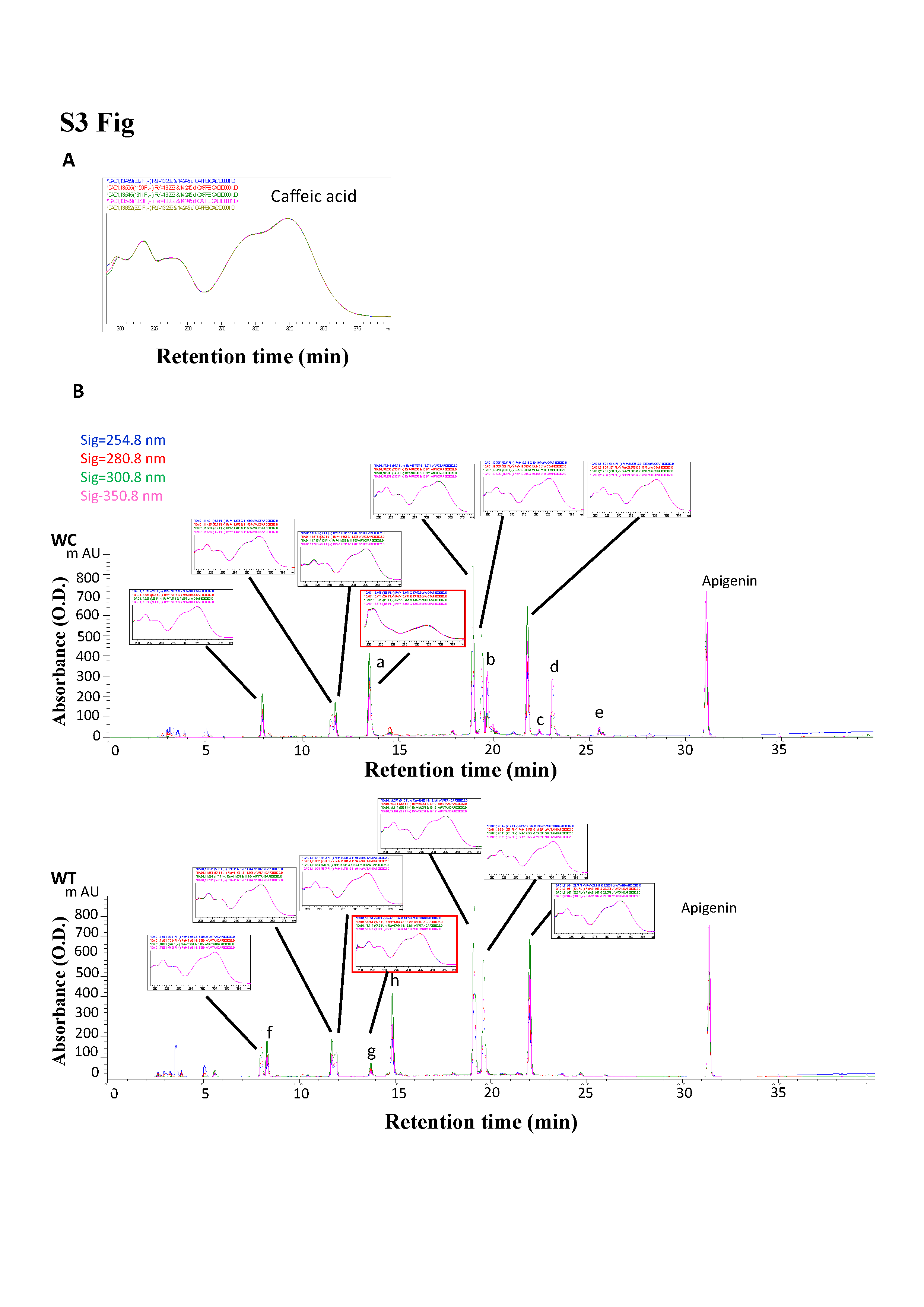

Supplement: S3 Fig — A, UV spectra detected for caffeic acid. B, UV spectra detected for some putative caffeic acid derivatives in the HPLC chromatograms of W. chinensis and W. trilobata. Apigenin is spiked in as the internal standard. (TIF) [file pone.0129067.s004.tif]

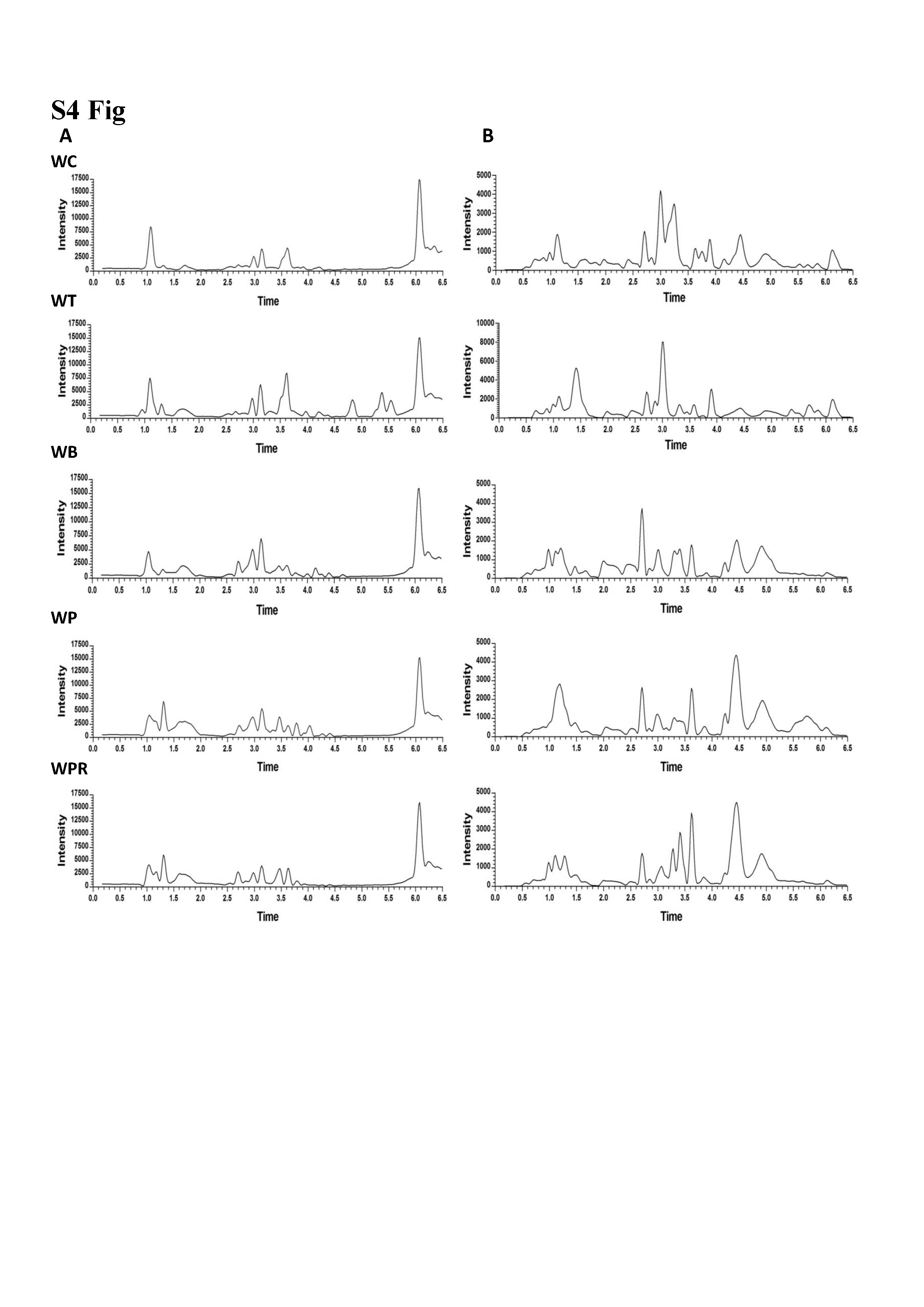

Supplement: S4 Fig — A, Electrospray ionization positive LC-MS data. B, Electrospray ionization negative LC-MS data. (TIF) [file pone.0129067.s005.tif]
