# Supplementary material for: Integrative Approach to Analyze Biodiversity and Anti-Inflammatory Bioactivity of Wedelia Medicinal Plants
Source: PLoS One. 2015 Jun 4;10(6):e0129067. doi: 10.1371/journal.pone.0129067 (PMC4456162; doi:10.1371/journal.pone.0129067)
Supplement: S1 Table — (DOCX) [file pone.0129067.s007.docx]

**S1 Table. Disease activity index.**

| Score | Weight loss | Stool consistency^a^ | Fecal blood |
| --- | --- | --- | --- |
| 0 | None | Normal | Normal |
| 1 | 1-5% | Normal | Normal |
| 2 | 5-10% | Loose stool | Hemoccult (+)^b^ |
| 3 | 10-20% | Loose stool | Hemoccult (+) |
| 4 | >20% | Diarrhea | Gross bleeding |

The disease activity index is the combined scores of body weight loss, stool consistency and fecal blood, resulting in a maximum DAI of 12. ^a^Normal stool = firm and well-formed pellets, loose stool = pasty and semi-formed stools that do not adhere to the anus, diarrhea = liquid stools that adhere to the anus. ^b^Hemoccult (+) = positive for fecal occult blood by using Hemoccult test (Beckman Coulter, Eschwege, Germany).
