# Supplementary material for: Integrative Approach to Analyze Biodiversity and Anti-Inflammatory Bioactivity of Wedelia Medicinal Plants
Source: PLoS One. 2015 Jun 4;10(6):e0129067. doi: 10.1371/journal.pone.0129067 (PMC4456162; doi:10.1371/journal.pone.0129067)
Supplement: S2 Table — (DOCX) [file pone.0129067.s008.docx]

**S2 Table. Scoring system for histological pathology study.**

| Organ/Histopathological finding | Histological score^a^ |
| --- | --- |
| Lost, crypt | 1-5 |
| Regeneration, crypt | 1-5 |
| Edema, submucosa | 1-5 |
| Inflammation, mononuclear cells | 1-5 |
| Ulcer, with fibroblast cell infiltration | 1-5 |

^a^Degree of lesion is graded from one to five depending on severity: 1 = minimal (< 1%); 2 = slight (1-25%); 3 = moderate (26-50%); 4 = moderate/severe (51-75%); 5 = severe/high (76-100%).
