# Supplementary material for: Integrative Approach to Analyze Biodiversity and Anti-Inflammatory Bioactivity of Wedelia Medicinal Plants
Source: PLoS One. 2015 Jun 4;10(6):e0129067. doi: 10.1371/journal.pone.0129067 (PMC4456162; doi:10.1371/journal.pone.0129067)
Supplement: S1 File — This is to certify that the animal protocol by the following applicant has been evaluated and approved by the Institutional Animal Care and Use Committee of Academia Sinica (AS IACUC). (PDF) [file pone.0129067.s006.pdf]

eProtocol Admin.
Dr. Fu-Hwa Liu.
Tel: (02) 2789-8700
iacuc@gate.sinica.edu.tw

Dear Dr. 楊, 寧蓀,

茲證明此動物實驗計畫書(如下列)業經中央研究院動物實驗管理委員會審核通過。

This is to certify that the animal protocol by the following applicant has been evaluated and approved by the Institutional Animal Care and Use Committee of Academia Sinica(AS IACUC).

Protocol ID: 11-10-232
Principal Investigator: **楊, 寧蓀**
Department: **農生中心**(ABRC)
Protocol Title: **建立可提供業界參與或主導的黃花蜜菜之抗腸炎/腸癌生物活性及相關功能之功能性食品**
Funding :**中研院**(Academia Sinica),Start Date : 01/01/2012, End Date :12/31/2012
Approval Date: 03/11/2014
Animal Species/Strains:Mouse / C57BL/6 (Inbred)
Number to be used per year: 200

Sincerely,
中央研究院動物實驗管理委員會召集人 孔祥智

John T. Kung Ph.D., Chair, AS IACUC

Please be noted that this protocol has to be renewed before 03/10/2015 for the purpose of internal monitoring.

e-protocol
